# Engineering the Coherent Phonon Transport in Polar Ferromagnetic Oxide Superlattices

**DOI:** 10.1002/advs.202407382

**Published:** 2024-11-21

**Authors:** In Hyeok Choi, Seung Gyo Jeong, Do‐Gyeom Jeong, Ambrose Seo, Woo Seok Choi, Jong Seok Lee

**Affiliations:** ^1^ Department of Physics and Photon Science Gwangju Institute of Science and Technology (GIST) Gwangju 61005 Republic of Korea; ^2^ Department of Physics Sungkyunkwan University Suwon 16419 Republic of Korea; ^3^ Department of Physics and Astronomy University of Kentucky Lexington Kentucky 40506 USA

**Keywords:** coherent phonon transport, polar phase transition, SrRuO_3_, SrTiO_3_, superlattice

## Abstract

Artificial superlattices composed of perovskite oxides serves as an essential platform for engineering coherent phonon transport by redefining the lattice periodicity, which strongly influences the lattice‐coupled phase transitions in charge and spin degrees of freedom. However, previous methods of manipulating phonons have been limited to controlling the periodicity of superlattice, rather than utilizing complex mutual interactions that are prominent in transition metal oxides. In this study on oxide superlattices composed of ferromagnetic metallic SrRuO_3_ and quantum paraelectric SrTiO_3_, phonon modulation by controlling the geometry of superlattice in atomic‐scale precision is realized, demonstrating the coherent phonon engineering using structural and magnetic phase transitions. By modulating the interface density, coherent‐incoherent crossover of the phonon transport at room temperature is observed, which is coupled with a change in interfacial structural continuity. Upon cooling, the close relation between phonon transport and multiple phase transitions is identified. In particular, the enhancement of the polar state in SrTiO_3_ layer at ≈200 K leads to the weakening of phonon coherence and a further reduction of thermal conductivity in superlattices compared to the bulk limit. These findings provide a guide to developing future thermoelectric nanodevices by engineering the coherence of phonons via the design of complex oxide heterostructures.

## Graphical abstract

Artificially‐designed perovskite oxide superlattices serves as an opportunity for engineering coherent phonon transport influenced by the lattice periodicity and phonon coherent length. A superlattice structure composed of ferromagnetic SrRuO_3_ and quantum paraelectric SrTiO_3_ provides the controllability of both the lattice periodicity and phonon coherent length modulated by the lattice‐coupled phase transitions in charge and spin degrees of freedom.

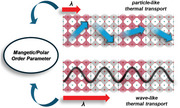

## 1 Introduction

Quantized lattice vibration modes in crystalline solids, i.e., phonons, determine the thermodynamic properties and promote emergent functional opto‐electronic and magnetic properties of materials by strongly coupling with charge and spin channels. Effective control of the phonons and their dynamics is crucial both for exploring the intriguing physical phenomena in solids and for facilitating their various applications. In particular, ultra‐low thermal conductivity *κ* can be achieved through phonon engineering.^[^ ^1^, ^2^, ^3^, ^4^, ^5^ ^]^ to realize the high figure of merit *ZT* of thermoelectric materials. However, because the characteristics of phonons are mainly prescribed by the crystalline lattice structure, actively manipulating them is challenging. Such control of phononic states has been achieved by fine‐tuning the structural parameters via, e.g., mechanical pressure, uniaxial or biaxial strain in epitaxial thin films, or isotope substitution.^[^ ^6^, ^7^, ^8^ ^]^ Under electronic and magnetic phase transitions, phononic states are usually modulated via interaction with the charge and spin degrees of freedom. For example, a commensurate charge order redefines the lattice periodicity, resulting in the modification of phonon dispersion.^[^ ^9^ ^]^ Upon a displacive‐type ferroelectric transition, a dramatic softening of optical phonons occurs via their direct coupling to the ferroelectric instability.^[^ ^10^ ^]^ In magnetically ordered phases, crystalline structures and the phonon states can be changed through magneto‐elastic coupling and/or spin‐phonon coupling.^[^ ^11^, ^12^ ^]^ These renormalizations of phononic states provide a viable strategy to modulate the dynamic properties of phonons and to realize useful thermodynamic functionalities.

**Figure 1:**
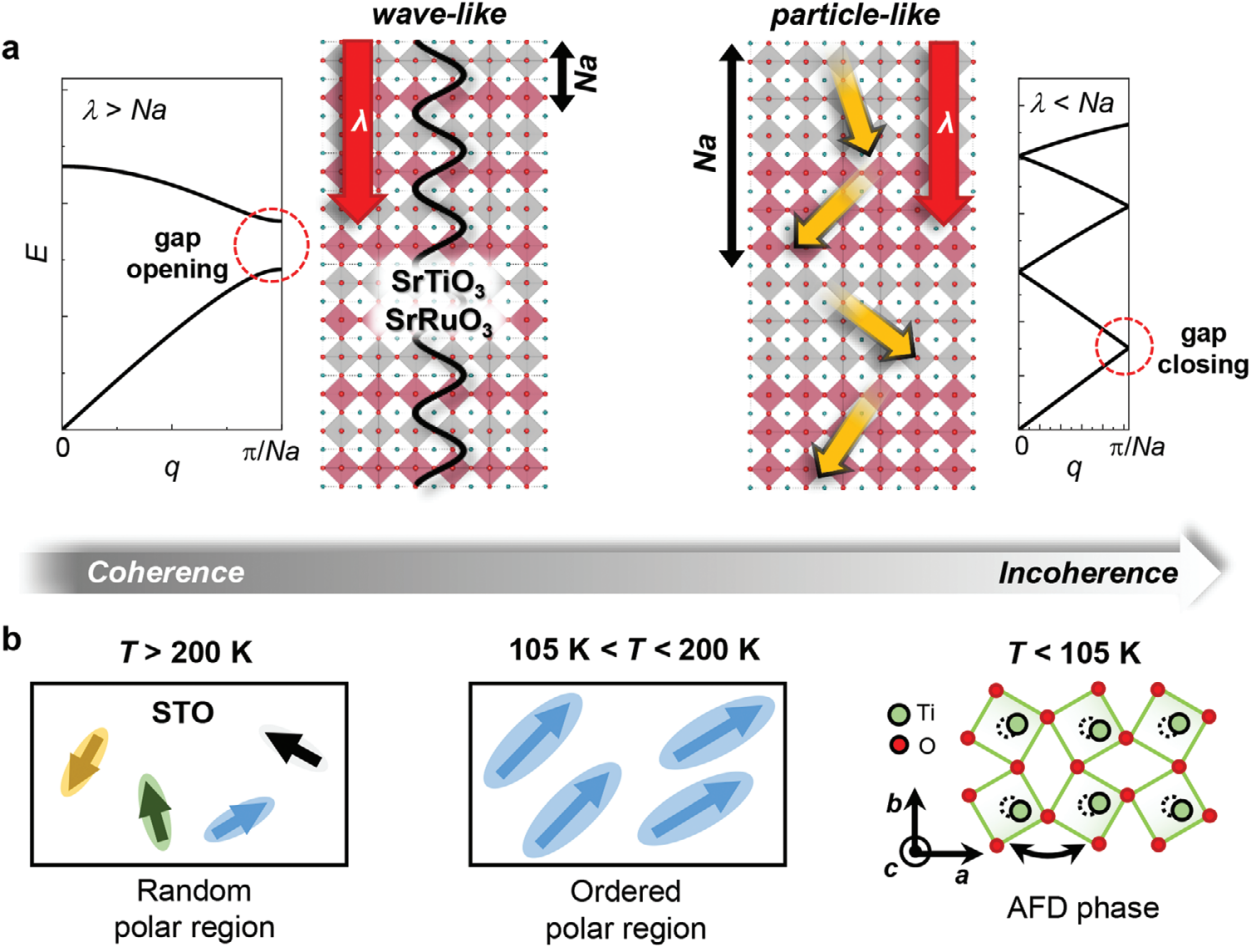
Coherent and incoherent phonon transport in SrRuO_3_/SrTiO_3_ superlattice (SRO/STO SL). a) Schematic descriptions of coherent and incoherent phonon transport. Left and right panels show that the two types of phonon transport are distinguished by the relative size of the phonon coherence length (*λ*) compared to the superlattice period (*Na*) in the SL composed of SRO (red) and STO (grey). For a given *Na*, these indicate a gap opening (*λ* > *Na*, left panel) and a gap closing (*λ* < *Na*, right panel) at the Brillouin zone center and boundary of the zone‐folded phonon dispersion for SRO/STO SLs. b) Temperature‐dependent structural phases of the STO layer in the SRO/STO SLs revealed by Raman spectroscopy and optical second‐harmonic generation measurement. These structure phase transitions can facilitate the manipulation of phonon transport in the SLs.

Recent advances in epitaxial film synthesis technology have provided a viable opportunity for engineering phononic states at the atomic scale. A superlattice (SL) structure composed of different materials artificially redefines the lattice periodicity with supercell thickness *Na*, where *N* is the number of unit cells, and *a* is the thickness of the unit cell. This naturally leads to zone‐folding of the phonon dispersion, resulting in the reduction of the Brillouin zone (BZ) to 2π/*Na*. At the reduced BZ, zone‐folded acoustic (ZA) phonons emerge,^[^ ^13^ ^]^ as shown in **Figure** **1**a, and their energy can be deliberately controlled by manipulating *N*. When *Na* is shorter than the phonon coherence length *λ* (*λ* > *Na*, coherent phononic state), gaps open at the BZ boundary due to wave interference, as schematically illustrated in the left panel of Figure 1a. The ZA phonons form a standing wave at the gap, leading to the reduction of *κ*; hence, *κ* decreases with increasing *Na* due to an increase in zone‐folding. On the other hand, if *Na* is much longer than *λ* (*λ* < *Na*, incoherent phononic state), ZA phonons lose their coherence due to the scattering process. This annihilates the wave interference of the ZA phonons at the BZ boundary, resulting in gap closure, as shown on the right panel of Figure 1a. Therefore, *κ* increases with increasing *Na* for *λ* < *Na* even if the number of zone‐folds increases. This indicates that *κ* of the SL can vary oppositely depending on the coherence (*λ* > *Na*) and incoherence (*λ* < *Na*) state of ZA phonons. When *Na* is equal to *λ*, *κ* becomes the minimum at the coherence‐incoherence crossover of the phonon. As the coherent nature of phonon transport in the SLs is determined by the relative length between *Na* and *λ*, this provides an opportunity to facilitate the engineering of *κ* both by changing *Na*.^[^ ^14^, ^15^, ^16^, ^17^, ^18^, ^19^ ^]^ with a geometrical design and by manipulating *λ* with phonon renormalization under appropriate electronic and magnetic phase transitions. A recent study for oxide SLs suggested the importance in designing both electronic part of thermal conductivity and the phonon spectrum to achieve high‐performance thermoelectric materials.^[^ ^20^, ^21^ ^]^ While previous studies have reported the realization of artificial phonons using SL structures, modulation of the artificial phonon using correlated electrical and/or magnetic phase transitions has rarely been reported.

Here, we demonstrate the manipulation of phononic properties in the SL composed of a quantum paraelectric SrTiO_3_ (STO) and a ferromagnetic metal SrRuO_3_ (SRO) to exploit lattice‐coupled phase transitions of each layer. By changing temperature *T* and/or strain, STO can exhibit several structurally distinct phases, including antiferrodistortive (AFD) and relaxor‐ferroelectric states containing nano‐sized polar regions.^[^ ^22^, ^23^, ^24^, ^25^, ^26^, ^27^ ^]^ On the other hand, SRO undergoes a ferromagnetic transition at ≈150 K accompanied by a change of the unit cell volume due to the spin‐phonon coupling.^[^ ^12^ ^]^ More recently, SRO/STO SL has been reported to host chiral phonons propagating across the SL.^[^ ^28^ ^]^ Therefore, the SRO/STO SL may exhibit *T*‐dependent multifunctional phases including relaxor‐ferroelectric and ferromagnetic phases, which are expected to modulate the phononic states within the SL. In particular, the emergence of polar cluster and phase transition into the AFD state in a relaxor‐ferroelectric state would modulate a phonon mean free path via polar/AFD domain scattering,^[^ ^23^ ^]^ providing additional controllability of phonon coherence in the SL (Figure 1b). Thus, the SRO/STO SL can serve as a valuable testbed to investigate how such correlated phase transitions influence phonon coherence and nanoscale thermal transport in SLs.

## 2 Results and Discussion

**Figure 2:**
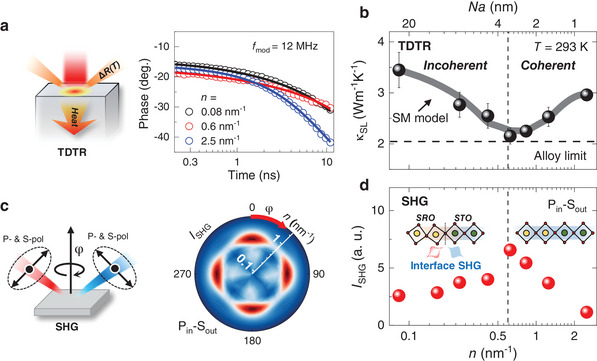
Coherence‐incoherence crossover of phonon transport in SRO/STO SLs at room temperature. a) Schematic of time‐domain thermo‐reflectance (TDTR) experiment (left panel) and raw data of the phase shift in the thermal response (right panel). By fitting experimental results based on the thermal model, we obtained both thermal conductivity *κ* and thermal boundary conductance *G*. b) Fitted κ for various interface densities *n*. There is a minimum value of *κ* at *n* = 0.7 nm^−1^ or the SL period *Na* = 2.8 nm, demonstrating coherence‐incoherence crossover of phonon transport. The black shaded line represents a theoretical expectation from the Simkin‐Mahan (SM) model. κ of SrRu_0.5_Ti_0.5_O_3_ solid solution is indicated with a dashed line (alloy limit). c) Schematic for the optical second‐harmonic generation (SHG) measurement in oblique geometry (left panel) with P/S_in_‐P/S_out_ polarization configurations, and SHG polar patterns for the SRO/STO SLs obtained with rotating sample azimuth (φ) and varying *n* in P_in_‐S_out_ configuration (right panel). d) Averaged SHG intensity in P_in_‐S_out_ configuration as a function of *n*. The SHG intensity shows the maximum at *n* ≈0.7 nm^−1^ where the minimum value of κ is observed. This demonstrates a close coupling between structural and phonon transport coherences, as schematically shown in the inset.

We first investigated the coherent phonon transport in SRO/STO SLs at room temperature (paramagnetic state). In describing the geometry of the SLs, we used the notation [*x*|*y*]*_z_*, where *x*, *y*, and *z* are the numbers of atomic unit cells (u.c., *a* ≈4 Å) of the SRO and STO layers and the total repetition, respectively. The total number of perovskite layers of all SLs was fixed at 120, i.e., ≈48 nm (confirmed by high‐resolution X‐ray diffractions as in Section S1, Supporting Information), and the interface density (*n* = 2/*Na*) was varied from 0.08 to 2.5 nm^−1^ by changing *x* (= *y*). To monitor the thermodynamic properties, we employed a time‐domain thermoreflectance (TDTR) technique, as schematically shown in **Figure** **2**a. By fitting the phase of thermal response at the surface (right panel of Figure 2a), we can sensitively determine *κ* of the SLs as well as the thermal boundary conductance (*G*) at the interface between the SRO and STO layers.^[^ ^29^ ^]^ (Section S2, Supporting Information). Figure 2b displays *κ* of the SLs as a function of *n* at room temperature. The *n*‐dependent *κ* exhibited a minimum at *n* ≈0.7 nm^−1^ (*Na* ≈2.8 nm), which is a clear signature of the crossover of phonon transport between the coherent and incoherent regimes. Here, *Na* ≈2.8 nm corresponds to *λ* of SRO/STO SLs, and it is similar to those of other oxide SLs.^[^ ^14^ ^]^ The minimum value of *κ* is ≈2.1 Wm^−1^ K^−1^, which is slightly larger than that of the alloy limit (Section S3, Supporting Information). The grey shaded line represents the calculated *κ* values with the Simkin‐Mahan (SM) model.^[^ ^30^ ^]^ considering the SL zone‐folding effect (Section S4, Supplementary Information), and it is well matched to our experimental result. We note that the entire thickness of SRO/STO SLs is much larger than the phonon mean free path of SRO (≈20 nm) and STO (≈2 nm),^[^ ^31^, ^32^ ^]^ and hence we can ignore the phonon confinement effect. Therefore, it is valid to interpret the thermal properties of our system with the SM model where the ideal phonon dispersion is calculated by adapting the periodic boundary condition. In the incoherent regime (*n* < 0.7 nm^−1^), the gaps at the BZ boundary, which are entirely closed at *n* = 0 (bulk limit), are gradually opened with increasing *n*, resulting in a decrease in *κ*. In contrast, *κ* increases with increasing *n* once the SL is within the coherent regime (*n* > 0.7 nm^−1^) due to the reduction of the number of SL zone‐folds as discussed previously. We note that potential charge transfer across the SRO/STO interface may lead to an electronic reconstruction, which might additionally contribute to the thermal conduction of the SL.^[^ ^20^ ^]^ However, the charge transfer between SRO and STO layers is known to be highly suppressed,^[^ ^28^, ^33^, ^34^, ^35^, ^36^ ^]^ and the STO layer remains insulating at least down to its two unit‐cell thickness.^[^ ^36^ ^]^ Consequently, we can rule out the additional electronic contribution to the cross‐plane thermal conduction in the SRO/STO SLs.

Interestingly, the coherence‐incoherence crossover of the phonon transport accompanies a change in the structural phase in SLs. The structural symmetry at the interface is monitored using a second harmonic generation (SHG) measurement, which provides information on the broken inversion symmetry. As schematically shown in Figure 2c, we set the input beam to be P‐polarized and detect the S‐polarized second‐harmonic beam (P_in_‐S_out_) rotating the sample azimuth (φ) in an oblique incidence. The SHG polar patterns of all SL exhibit four‐fold symmetry (right panel of Figure 2c), which is well explained by a combination of two orthogonal non‐centrosymmetric monoclinic domains expected for the SRO/STO interface.^[^ ^37^ ^]^ (Section S5, Supporting Information). Figure 2d displays a φ‐averaged intensity of the SHG signal as a function of *n*. The averaged SHG intensity shows a maximum value at *n* ≈0.7 nm^−1^, which is the same as that observed in phonon transport (Figure 2b). In the incoherent regime, the SHG intensity increases with the number of SRO/STO interfaces. As SRO and STO layers exhibit distinct octahedral tilting angles,^[^ ^33^ ^]^ a structural discontinuity at the interface can be the origin of the SHG signal. In the coherent regime (*n* > 0.7 nm^−1^), however, the SHG intensity decreases with increasing *n*, demonstrating enhanced interfacial structural coherence. As the layer thicknesses of SRO become atomically thin (a few unit cells), the SRO layers undergo a structural change from orthorhombic (at bulk) to tetragonal phase (at thin film) within the SL, resulting in structural coherence with the same octahedral tilting angle across the interface. This correlation between the structural symmetry and *κ* suggests that the structural coherence‐incoherence crossover in SRO/STO SLs can have a large influence on the coherence length of structural dynamics, namely phonon transport.^[^ ^38^ ^]^

**Figure 3:**
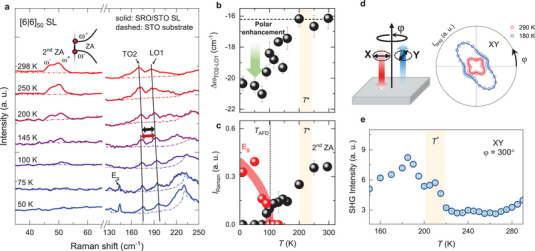
Temperature‐dependent Raman spectroscopy results in [6|6]_50_ SL. a) The second zone‐folded acoustic (2nd ZA) phonon and STO ferroelectric phonons obtained at various temperatures *T*. The 2nd ZA phonon was observed in the SL at 49 cm^−1^, splitting into upper and lower modes, denoted by *ω* ^+^ and *ω* ^−^, respectively. STO ferroelectric phonons TO2 and LO1, which are Raman‐active modes in the non‐centrosymmetric structure, were clearly observed at 170 and 186 cm^−1^, respectively (black solid line). Below 100 K, *E*_g_ phonon mode arose at 144 cm^−1^, representing the antiferrodistortive (AFD) phase transition in the STO layer. b) *T*‐dependent peak position difference between LO1 and TO2 phonons, and c) Raman peak intensity of *E*_g_ and 2nd ZA phonon modes. From these results, two distinct structural transition temperatures were determined as *T* ^*^≈200 K (shaded area) and *T*_AFD_≈100 K (dashed line). d) φ‐dependent SHG polar patterns measured at 290 and 180 K with orthogonal polarizations between input and SHG beams (XY) in normal incidence, and e) their intensity obtained at φ = 300° as a function of *T*. The SHG polar patterns were fitted by considering two orthogonal monoclinic domains.

To further investigate the correlation between the structural phases and phonons, we characterized *T*‐dependent structural phases of the SLs using confocal Raman spectroscopy.^[^ ^39^, ^40^, ^41^ ^]^ Here, we chose the [6|6]_50_ SL, which has each layer thickness comparable to *λ* (≈2.8 nm). We obtained Raman spectra of both the SL and the STO substrate by precise control of the out‐of‐plane‐directional (confocal) beam position. **Figure** **3**a shows Raman spectra for the SL (solid line) and the STO substrate (dashed line) at several *T*. At room temperature, two phonons observed at ≈170 and 186 cm^−1^ correspond to the transverse optical (TO2) and longitudinal optical (LO1) excitations of ferroelectric STO thin film phases, respectively.^[^ ^42^, ^43^, ^44^, ^45^ ^]^ Note that a single crystalline STO bulk substrate does not show the phonon excitations due to its centrosymmetric structure (Section S6, Supporting Information). As these ferroelectric phonons are Raman‐active in the non‐centrosymmetric system,^[^ ^44^ ^]^ their presence reveals the polar nature of the SLs, consistent with the SHG result. This can be understood from the finding that thin STO layers can host broken inversion symmetry by local strain, as discussed in several previous studies.^[^ ^25^, ^26^, ^27^, ^46^, ^47^ ^]^

By tracing these phonon modes upon cooling, we identify the *T*‐dependent evolution of structural phases of SRO/STO SLs. First, we note an intermediate temperature *T* ^*^ » 200 K associated with the development of the local polarization.^[^ ^47^ ^]^ As shown in Figure 3a, there seems to be an anomalous change in the frequency of the TO2 phonon *ω*_TO2_ as *T* decreases from 200 to 145 K (guided by black lines). We monitor the *T*‐dependent frequency difference between TO2 and LO1 phonons, namely Δ*ω* = *ω*_TO2 –_ *ω*_LO1_, where *ω*_TO2_ and *ω*_LO1_ are obtained by fitting with Lorentzian functions (as summarized in Figure 3b, Section S7, Supporting Information). As the LO1 mode follows a monotonic *T*‐dependent frequency shift due to the phonon anharmonic effect,^[^ ^48^ ^]^ Δ*ω* can highlight the *T*‐dependent frequency shift for the TO2 mode, related to the polarization field.^[^ ^49^ ^]^ *T* ^*^ is defined as ≈200 K when Δ*ω* starts to decrease. The apparent softening of TO2 phonons below *T* ^*^ can be related to the development of the polar nano‐regions, which has been reported in relaxor‐ferroelectric perovskite oxides.^[^ ^47^, ^50^, ^51^, ^52^, ^53^, ^54^ ^]^ *T*‐dependent SHG results also support this assignment of the structural phases. Figure 3d displays SHG polar patterns obtained at 290 and 180 K in normal incidence, providing information on the in‐plane polarization (Section S8, Supporting Information). Notably, the isotropic four‐fold SHG polar pattern at 290 K (red) changed into the two‐fold‐like polar pattern at 180 K (blue) with increasing intensity, demonstrating the enhancement of in‐plane polarization. The *T*‐dependent SHG intensity obtained at φ = 300° clearly exhibits a structural phase transition at *T* ^*^ (Figure 3e), supporting the existence of the long‐range polar order in SRO/STO SLs below *T* ^*^. Nevertheless, we cannot conclude whether the entire STO layer would have similar ferroelectric‐like displacements, and also whether the STO layer has actual ferroelectric states. Second, we can define the AFD phase transition temperature (*T*_AFD_) at ≈100 K. As shown in Figure 3a, an additional phonon peak appeared at ≈144 cm^−1^ below 100 K. This mode is attributed to the *E*_g_ phonon, which is clear evidence of the structural phase transition into the AFD phase of STO (both the bulk and the film).^[^ ^53^, ^55^ ^]^

In the Raman spectrum at room temperature, an additional peak is observed at ≈49 cm^−1^ for the SRO/STO SL, which is absent for the STO substrate. This corresponds to the 2nd ZA phonon arising from the zone‐folding in the SL.^[^ ^13^ ^]^ The peak is split into lower (*ω* ^−^) and upper (*ω* ^+^) ZA phonons, demonstrating the existence of the phonon band gap near the BZ boundary. As shown in Figure 3a,c, the intensity of the 2nd ZA phonon significantly decreased with decreasing *T* below *T* ^*^, and the peak was absent below *T*_AFD_. This diminishing ZA phonon peak demonstrates a weakening of the SL phonon coherence, which should be attributed to the additional structural decoherence originating from correlated electron and/or spin phase transitions upon cooling.

**Figure 4:**
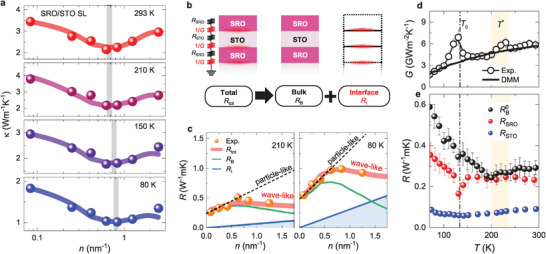
Temperature‐dependent thermal transport in SRO/STO SLs under complex structural phases. a) *n*‐dependent κ of SRO/STO SLs at various *T*. The gray lines indicate the interface density *n* that shows the minimum value of *κ*. Colored shaded lines represent theoretical expectations from the Simkin‐Mahan (SM) model at each *T*. b) Schematic of the series thermal resistance model and c) fitting results for thermal resistance *R* (= 1/*κ*) at 210 and 80 K with the SM model. Red, green, and blue lines represent total *R* (*R*_tot_), bulk (*R*_B_), and interface (*R*_I_) contributions, respectively. d) *T*‐dependent thermal boundary conductance *G*. We compared the experimental result with that obtained from the diffuse mismatch model (DMM) depicted by a black solid line. e) *T*‐dependent *R*_B_ at *n* = 0 (*R*_B_^0^) and its comparison with those of SRO (*R*_SRO_) and STO (*R*_STO_). Note that *R*_SRO_ was obtained from the 32‐nm‐thick SRO film, the thickness of which is comparable to that of SLs (48 nm). *R*_STO_ was obtained from the STO substrate in which the thermal conductivity was assumed to remain the same with a thickness reduction at least down to 48 nm.

To elucidate how the transport of phonons is influenced by the phase transition, we monitored the *T*‐dependent *κ* of the SLs (Section S9, Supporting Information). **Figure** **4**a displays *n*‐dependent variations of *κ* at four representative *T* (80, 150, 210, and 293 K); the minimum value of *κ* was observed at all *T*. In general cases, the phonon mean free path closely correlated with *λ* increases with decreasing *T* due to a reduction of the scattering rate.^[^ ^56^, ^57^ ^]^ This results in a decrease in *n* exhibiting the minimum value of *κ* with decreasing *T*, as previously reported in SrTiO_3_/CaTiO_3_ SLs.^[^ ^14^ ^]^ Contrary to this expectation, the *n* value of the minimum *κ* in the SRO/STO SLs systematically increased with decreasing *T* over all observed *T‐*regions. In addition to the loss of ZA phonons below *T* ^*^, this result demonstrates that phonon transport is largely influenced by the reduced phonon coherence under the correlated phase transition, which is distinct from previous studies.^[^ ^14^, ^15^, ^16^, ^17^, ^18^, ^19^ ^]^

To obtain further insight, we examined *κ* of the SLs based on a series thermal resistance model. As shown in Figure 4b, the interface contribution to the thermal resistivity *R*_I_ was separately considered together with the bulk contribution *R*_B_, whereas the thermal resistivity (*R* = 1/*κ*) of the SLs was given by their linear sum. In particle‐like thermal transport, it is assumed that all phonon modes are incoherent, and hence there is no gap opening at the reduced Brillouin zone boundary. As a result, *R*_B_ is independent of *n*, and *R*_I_ is given by *n*/*G*, where *G* is the thermal boundary conductance across the SRO/STO interface. In wave‐like thermal transport, on the other hand, *R*_B_ can be manipulated by changing *n* and can be calculated by the SM model. In Figure 4c, we display the fitting results for the *n*‐dependent *R* of SLs at 210 and 80 K with two fitting parameters *R*_B_ and *G*. While the particle‐like model (dashed line) explains the behavior only at low *n*, the wave‐like model (red shade line) successfully reproduced the experimental results in the entire range of *n*. Colored shaded lines in Figure 4a indicate the calculated *κ* with the wave‐like model at different *T*, which are in good agreement with the experimental results. Both *R*_B_ (green line) and *R*_I_ (blue line) exhibit much larger values at 80 K than at 210 K, which is attributed to a reduction of the number of phonons at the lower *T*.

We now examine the *T*‐dependent behaviors of *G* and *R*_B_ obtained from the fitting analyses using the SM model. Figure 4d displays *G* as a function of *T*. At room temperature, *G* is ≈6 GWm^−2^ K^−1^, which is a typical value for the atomically well‐defined oxide interface.^[^ ^14^, ^58^, ^59^ ^]^ The overall *T*‐dependent behavior of *G* is well described by a prediction based on the diffuse mismatch model (black solid line). However, there are large deviations around *T* ^*^ and the ferromagnetic transition temperature of the SRO layer (*T*_c_ ≈130 K), which is similar for SRO/STO SLs studied in this work.^[^ ^28^, ^60^ ^]^ The enhancement of *G* at *T* ^*^ and *T*_c_ demonstrates that both electrical polar and ferromagnetic order are related with phonon transport in the SL. Note that *G* does not exhibit any notable change at *T*_AFD_, consistent with the experimental result of the bulk STO (Sections S2, S10, Supporting Information).

Importantly, the bulk thermal transport in SLs also exhibits anomalous behaviors upon the emergence of local polarization. Figure 4e shows the *T*‐dependent *R*_B_ (black symbol) at the zero‐interface density, named *R*_B_^0^, which is taken from the SM model prediction at *n* = 0. In a simple expectation, *R*_B_^0^ is given as an average of *R* of SRO (*R*_SRO_) and STO (*R*_STO_).^[^ ^30^ ^]^ Figure 4e displays a comparison of *R*_B_^0^ with *R*_SRO_ and *R*_STO_. Here, *R*_SRO_ is the resistivity measured for the 32‐nm‐thick SRO thin film having a similar thickness to the SL, and *R*_STO_ is that of the STO substrate. As the phonon mean free path of STO is ≈2 nm,^[^ ^32^ ^]^ a finite thickness effect would not be significant for the STO film with a 32 nm thickness, which can be assumed to have a similar *R* to its bulk. At room temperature, *R*_B_^0^ is ≈0.3 W^−1^ mK, which is comparable to *R*_SRO_ but much larger than *R*_STO_. Below *T* ^*^, *R*_B_^0^ rapidly increases, while there is no notable change in either *R*_SRO_ or *R*_STO_, resulting in enhancement of *R*_B_^0^ by ≈50% compared to *R*_SRO_ at 80 K. This implies that formation of polar nano‐regions below *T* ^*^, which can be an efficient scattering source,^[^ ^61^, ^62^ ^]^ leads to a reduction of both the phonon coherence length *λ* and the phonon mean free path determining *R*_B_^0^ in the SRO/STO SLs. Importantly, this validates our original conjecture that the coherent nature of the phonon transport in the SL can be adjusted by controlling the structural phases. For SRO/STO SLs, the reduction of *λ* upon polar state formation results in the additional reduction of *κ* at high *n*, where wave‐like phonon transport is dominant.

## 3 Conclusion

In summary, we demonstrated the manipulation of coherent phonon transport under the phase transitions in SLs composed of ferromagnetic metallic SRO and quantum paraelectric STO. At room temperature, the SRO/STO SL exhibits coherent‐incoherent crossover of phonon transport, correlated with its structural coherence across the interfaces. As *T* decreases, we identified two structural phase transitions in the SRO/STO SL using Raman spectroscopy and SHG experiments. In addition to the ferromagnetic transition of SRO layers, multiple structural and magnetic phase transitions of SRO/STO SLs revealed intriguing *T*‐dependence of phonon transport. Such phase transitions can reduce both the phonon coherence length and phonon mean free path, weakening phonon coherence and possibly facilitating ultra‐low *κ* even at low *T*. Our findings demonstrate a novel approach to control the phonon transport in SLs via the correlation between ZA and optical phonons and other degrees of freedom in the course of the phase transitions. As the phase transitions can be driven, for example, by light irradiation, current flow, and applications of electric and/or magnetic fields, it is highly feasible to manipulate the coherent thermal transport by external stimuli.

## 4 Experimental Section

### Pulsed Laser Epitaxy

Atomically designed SRO/STO SLs were grown by pulsed laser epitaxy using a KrF excimer laser (248 nm wavelength, IPEX864, LightMachinery) on single‐crystalline STO (001) substrates. Before growth, the substrate to achieve a single‐terminated TiO_2_ was chemically treated and atomically flat surface using buffered oxide etchant (BOE). The substrate temperature was maintained at 750 °C, and a laser fluence of 1.5 J cm^−2^ with a frequency of 5 Hz was used. Stoichiometric ceramic SRO and STO targets were utilized, with a dynamic oxygen pressure of 100 mTorr to obtain stoichiometric SRO and STO layers. The number of unit cells (u.c.) of the SLs was controlled using a customized automatic laser pulse control system programmed with LabVIEW. The periodicity of the SLs was adjusted by varying the number of laser pulses for each of the SRO and STO layers with different repetitions. The atomically well‐defined structure of the epitaxial SLs was characterized by high‐resolution X‐ray diffraction (PANalytical X'Pert XRD),^[^ ^28^, ^33^, ^34^, ^35^, ^36^ ^]^ as shown in Figure S1 (Supporting Information).

### Time‐Domain Thermoreflectance (TDTR) Measurement

The TDTR technique to measure the thermal conductivity κ of SLs and the thermal boundary conductance *G* defined at the interface between the SRO and STO layers was utilized.^[^ ^31^ ^]^ For all the samples, Al layers were deposited as a transducer, and their thicknesses were determined by acoustic echoes at ≈70 nm. A pulsed laser with an 80 MHz repetition rate (Vision‐S, *Coherent*) was employed. Both pump and probe laser beams were tightly focused with a beam size of 30 µm at 1/*e* ^2^. The pump beam was modulated by an electro‐optic modulator at 12 MHz, and the pump‐induced reflectivity change using a high‐bandwidth photodiode detector (*Thorlabs*, Inc.) connected to a digital lock‐in amplifier (*Zurich*) was measured. Both pump and probe beams have center wavelengths of 785 nm, so it was adopted the two‐tint method to eliminate the pump signal in the detector. Since the cross‐plane thermal penetration depth of the heat source was calculated as ≈0.21 µm, being much smaller than the beam size, the contribution of in‐plane thermal transport was negligible.^[^ ^29^ ^63^, ^64^, ^65^ ^]^

### Optical Second‐Harmonic Generation (SHG) Measurement

The SHG experiment in both normal and oblique incidence geometries by rotating the sample azimuth (φ) was conducted. The input beam was focused with a beam size of 30 µm at 1/*e* ^2^, and its power was ≈40 mW. In the oblique incidence, the input beam was set to be P‐ or S‐polarized, and it was monitored P‐ or S‐polarized SHG signals. In the normal incidence, the SHG signal with two polarization configurations was measured, parallel (XX) and orthogonal (XY) polarizations between input and SHG beams. The SHG signal was measured using a photomultiplier detector (*Hamamatsu*), amplified with a current pre‐amplifier (*SRS*). To eliminate the fundamental signal before it reaches the detector, short‐pass and band‐pass filters were used.

### Confocal Raman Spectroscopy

Raman spectra of [6|6]_50_ SL were obtained using a confocal micro‐Raman spectrometer (Horiba LabRam HR800) equipped with a 632.8 nm (1.96 eV) HeNe laser. Measurements were conducted on SRO/STO SL with 50 repetitions (total thickness of ≈240 nm) to enhance the inelastic light scattering cross‐section to detect Raman scattering of each atomically thin SRO and STO layer. *T*‐dependent measurements were performed in a vacuum using a cold‐finger‐type optical cryostat. A grating with 1800 grooves per mm and a focused beam spot of 5 µm were used. Laser power was kept below 0.3 mW to avoid laser heating effects. Precise control of the *z*‐directional beam position ensured optimal focus on the SL samples, enabling the acquisition of high‐quality Raman spectra in the backscattering geometry.^[^ ^13^, ^28^ ^]^

## Conflict of Interest

The authors declare no conflict of interest.

## Author Contributions

I.H.C. and S.G.J. contributed equally to this work. J.S.L. and W.S.C. supervised this work. I.H.C., D.G.J., and J.S.L. performed time‐domain thermoreflectance measurements. I.H.C. and J.S.L. performed the second harmonic generation, and analyzed the data. I.H.C. and D.G.J. conducted the numerical calculations. S.G.J. and W.S.C. prepared superlattices and characterized them. S.G.J., A.S., and W.S.C. conducted Raman spectroscopy experiments, and analyzed data. I.H.C., S.G.J, W.S.C., and J.S.L. wrote the manuscript. All the authors discussed the results, and commented on the manuscript.

## Supporting information



Supporting Information

## Data Availability

The data that support the findings of this study are available from the corresponding author upon reasonable request.
