# Supplementary material for: Engineering the Coherent Phonon Transport in Polar Ferromagnetic Oxide Superlattices
Source: Adv Sci (Weinh). 2024 Nov 21;12(2):2407382. doi: 10.1002/advs.202407382 (PMC11727387; doi:10.1002/advs.202407382)
Supplement: Supplementary file 1 — Supporting Information [file ADVS-12-2407382-s001.docx]

**Supporting Information**

**Engineering the Coherent Phonon Transport in Polar Ferromagnetic Oxide Superlattices**

In Hyeok Choi^1,☨^, Seung Gyo Jeong^2,☨^, Do-Gyeom Jeong^1^, Ambrose Seo^3^, Woo Seok Choi^2,*^, and Jong Seok Lee^1,*^

^1^*Department of Physics and Photon Science, Gwangju Institute of Science and Technology (GIST), Gwangju 61005, Republic of Korea*

^2^*Department of Physics, Sungkyunkwan University, Suwon 16419, Republic of Korea*

^3^*Department of Physics and Astronomy, University of Kentucky, Lexington, Kentucky 40506, United States*

^☨^ Equally contributed

^*^Corresponding authors: [choiws@skku.edu](mailto:choiws@skku.edu), [jsl@gist.ac.kr](mailto:jsl@gist.ac.kr)

**The table of contents**

S1. X-ray diffraction pattern

S2. Time-domain thermoreflectance measurement for SrRuO_3_/SrTiO_3_ superlattices

S3. Thermal conductivity of SrRu_1-x_Ti_x_O_3_ solid solution

S4. Simkin-Mahan model

S5. Second harmonic generation measurements in oblique geometry for SrRuO_3_/SrTiO_3_ superlattices

S6. Raman spectrum at room temperature

S7. Fitting results for phonon modes with Lorentzian distributions

S8. Temperature-dependent second harmonic generation in normal incidence

S9. Temperature-dependent thermal conductivity of SrRuO_3_/SrTiO_3_ superlattices

S10. Temperature-dependent thermal conductivity of the SrTiO_3_ substrate

**S1. X-ray diffraction pattern**

**Figure S1. X-ray diffraction (XRD) diffraction patterns of SRO/STO SLs with various periods.**

We characterized the structural properties of SrRuO_3_/SrTiO_3_ superlattices (SRO/STO SLs) with various period thicknesses using the X-ray diffraction measurement. As shown in Fig. S1, we clearly observed Laue oscillations for all samples, demonstrating that films are well grown. Furthermore, SL satellite peaks (colored arrows) are systemically changed with respect to the period thickness, and they show the atomically well-defined interface between SRO and STO.

**S2. Time-domain thermoreflectance measurement for SrRuO_3_/SrTiO_3_ superlattices**


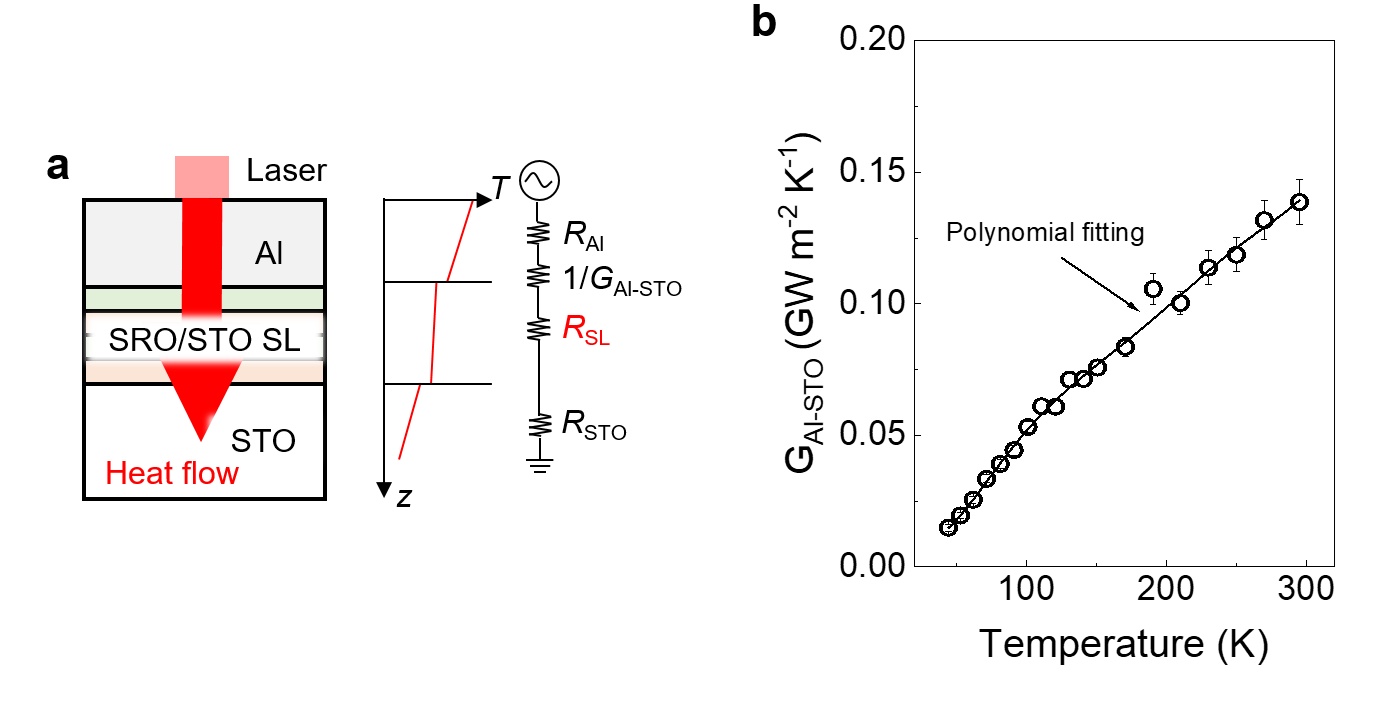


**Figure S2. Thermal conductivity measurement for SRO/STO SLs using time-domain thermoreflectance (TDTR) technique. (a)** Schematic of TDTR experiment for measuring κ of SLs. **(b)** Thermal boundary conductance between Al and STO with varying temperature.

Time-domain thermoreflectance (TDTR) technique can sensitively measure not only the thermal conductivity κ of nanoscale thin film but also the thermal boundary conductance *G*. In the complex heterostructure, however, there are various sources of thermal resistance, as shown in Fig. S2a, and accordingly the fitting sensitivities to thermal parameters become reduced. To secure the sensitivity for κ of SLs, we pre-determined the other thermal parameters, such as κ of Al and *G* of Al-STO interface (*G*_Al-STO_). First, we estimated κ of Al by simply converting the electric conductivity using the Wiedermann-Franz law. Second, we determined *G*_Al-STO_ from the TDTR measurement for the STO substrate. Since our SRO/STO SLs are terminated by the STO layer, *G* between Al transducer and the SL is same to *G*_Al-STO_. Figure S2b shows the measured *G*_Al-STO_ as a function of temperature. We use these pre-determined values to fit the temporal thermoreflectance profiles measured in the SLs with the two fitting parameters, namely thermal conductivities of the SL and the STO substrate.

**S3. Thermal conductivity of SrRu_1-x_Ti­_x_O_3_ solid solution**


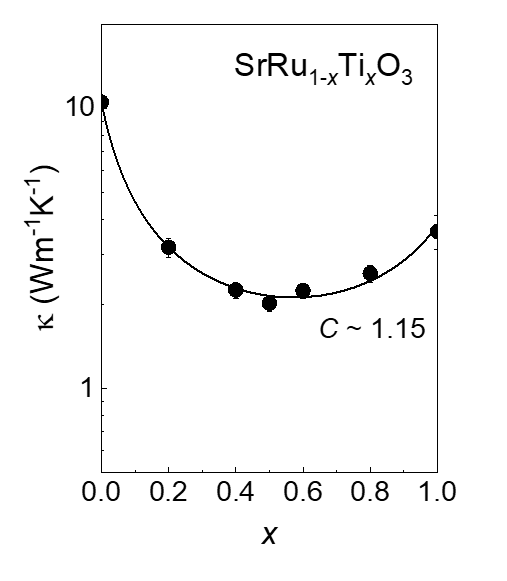


**Figure S3. Thermal conductivity of SrRu_1-x_Ti_x_O_3_ solid solution at room temperature.**

To compare the minimum value of κ in SLs and its alloy limit, we measured the κ of 32 nm thick SrRu_1-x_Ti_x_O_3_ films in a form of the solid solution on the STO substrate. Figure S3 displays the thermal conductivity determined at room temperature. The minimum κ is clearly observed around *x* = 0.5, exhibiting the maximum alloy-disorder scattering rate. We fit the κ of solid solutions with the theoretical model suggested by Adachi ^[1]^, and it is in good agreement in the experiment result. A disorder parameter *C* is given by 1.15, which is much smaller than other semiconductor-based SLs ^[1]^.

**S4. Simkin-Mahan model**


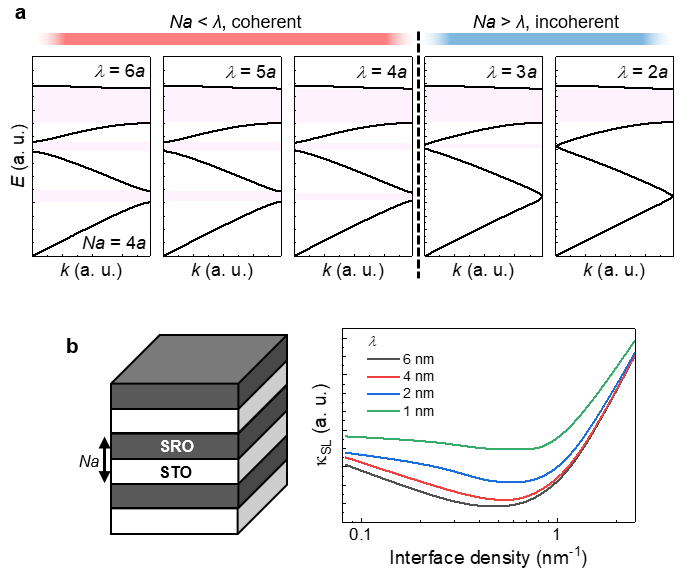


**Figure S4. Calculated thermal conductivity κ for various λ with Simkin-Mahan (SM) model considering the wave interference effect.**

To describe the thermal transport in SLs, a coherent phonon transport should be considered. The Simkin-Mahan (SM) model describes the thermal transport considering the SL zone-folding effect based on the wave theory ^[2]^. This minimal model employs the simple diatomic chain model to calculate the phonon band in the SL with the periodic boundary condition. We calculated the 3D phonon band with considering 3D phonon wave vectors (*q_x_*_,_ *q_y_*, *q_z_*) and set the mass ratio between the SRO and STO as 1.77. To implement the effect of phonon coherence length λ, the complex part *i*/λ is added to wave vector. Figure S4a exhibits the calculated phonon band structure with changing λ from the 6*a* to 2*a*, where *a* is a lattice constant and periodic thickness *Na* is 4*a*. In the coherent regime (*Na* < λ), gaps are opened at all of the Brillouin zone boundary due to the wave interference effect, where the phonon group velocity is zero. In the incoherent regime (*Na* > λ), however, the gap starts continuously closing from the lowest energy value with decreasing λ. This leads to an increase in thermal conductivity κ that is proportional to the sum of phonon group velocity. Figure S4b displays the calculated κ as a function of interface density (= 2/*Na*) with varying λ; the minimum value of κ is clearly observed, demonstrating the coherent phonon transport in the SL. The interface density that shows the minimum κ decreases with decreasing λ, which is consistent to our expectation.

**S5. Second-harmonic generation measurements in oblique geometry for SrRuO_3_/SrTiO_3_ superlattices**


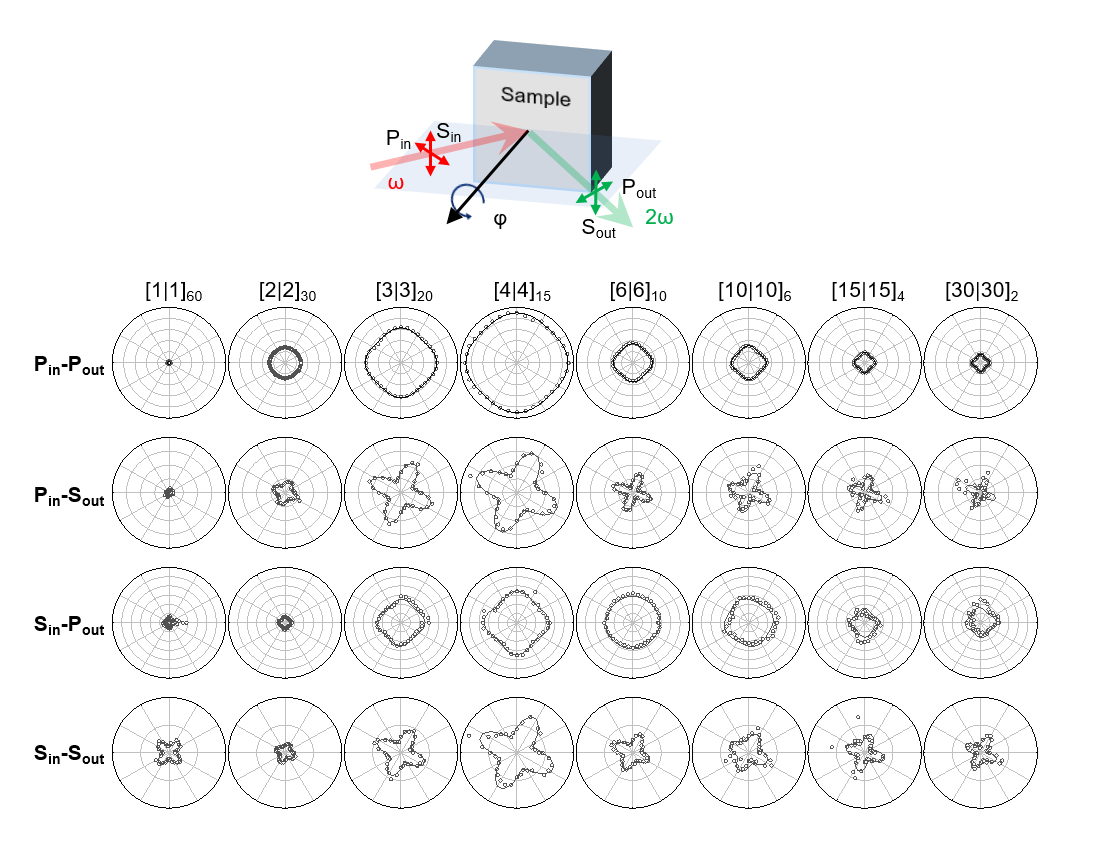


**Figure S5. Rotational anisotropy of the second harmonic generation intensity for SRO/STO SLs with varying the period thickness in the oblique geometry.**

Figure S5 displays the thickness-dependent rotational anisotropy patterns of second harmonic generation (SHG) for various polarization configurations. In this measurement, we set the input fundamental beam as P- or S-polarized (P_in_ or S_in_) and monitored the P- or S-polarized second harmonic beam (P_out_ or S_out_). Whereas all the samples have the same total thickness, the SHG signal exhibits thickness-dependent variations. This demonstrates that the observed SHG response does not originate from either the STO substrate or the bulk quadrupole contribution of the SLs which have same thickness. Instead, we consider the electric dipole contribution, induced by the inversion symmetry breaking at the SRO/STO interface, can be the major source of the SHG signal. In all polarization configurations, four-fold anisotropic patterns are clearly observed. Since the SHG signal in the S_in_-S_out_ configuration cannot be allowed in both orthorhombic and tetragonal symmetries, we consider the lower symmetry to explain the observed anisotropy patterns. We found that the fitting with the monoclinic *m* symmetry (solid lines) can explain the four-fold anisotropic patterns as well as the responses in the S_in_-S_out_ configuration.

**S6. Raman spectrum at room temperature**


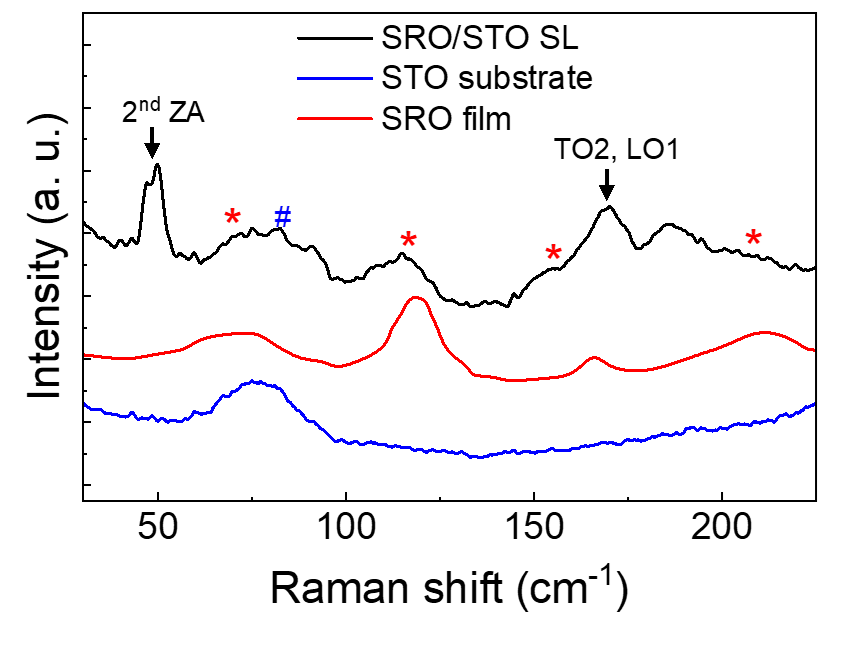


**Figure S6. Raman spectra for the [6|6]_50_ SL, SRO film and STO substrate at room temperature.** Phonon modes in the SL, SRO and STO are represented by black arrows, red (*) and blue (#) symbols, respectively.

The Raman spectrum of the SRO/STO SL includes the contributions of not only SL but also the STO and SRO layers. In Fig. S6, we compare the Raman spectra of the [6|6]_50_ SL with those of the SRO film and the STO substrate, and found the signals solely attributable to the SL itself. For the STO substrate, there exhibits only one phonon mode at 80 cm^-1^, corresponding to the polar TO1 phonon mode. For the SRO film, there are four peaks observed at 70 cm^-1^, 117 cm^-1^, 166 cm^-1^ and 210 cm^-1^, which are Raman-active A_g_ and B_g_ modes ^[3]^. We indicate the phonon modes from SRO and STO as red (*) and blue (#) symbols, respecteviely. For the SL, there appear several peaks which are absent in both SRO and STO results. A peak at 49 cm^-1^ is attributed to SL zone-folded acoustic (ZA) phonons. And, two peaks at 170 cm^-1^ and 186 cm^-1^ are ascribed to STO ferroelctic phonon modes TO2 and LO1, respectively. Since they are Raman-active only in a non-centosymmetric structure, their presence demonstrates the polar nature of the SRO/STO SL.

**S7. Fitting results for phonon modes with Lorentzian distributions**


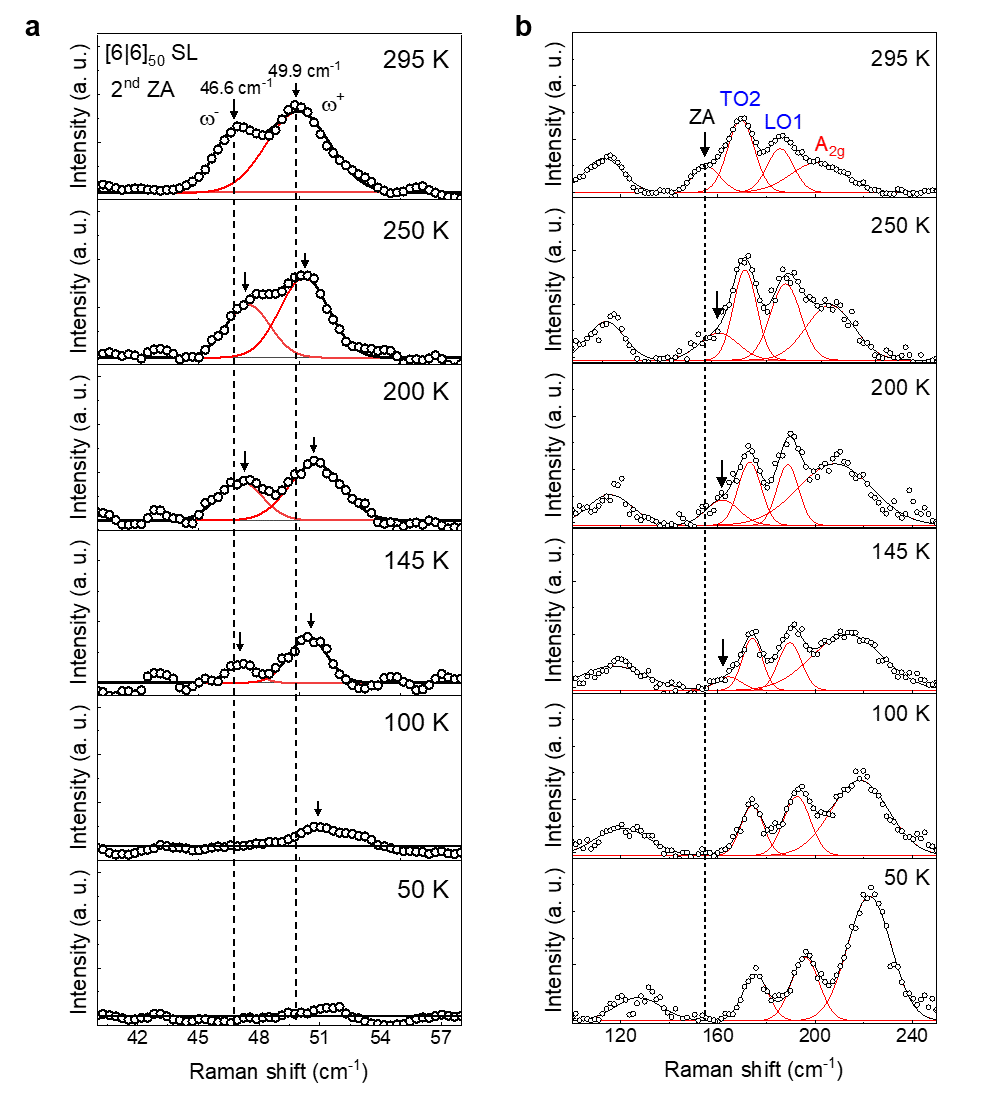


**Figure S7. Temperature-dependent 2^nd^ ZA and STO ferroelectric phonon modes. (a)** 2^nd^ ZA phonon fitted with two Lorentzian distributions. ω^+^ and ω^-^ represent the lower and upper ZA phonon, respectively. **(b)** Fitting results for the Raman spectrum from 110 cm^-1^ to 240 cm^-1^.

To analyze the results more quantitatively, we fit 2^nd^ ZA and STO ferroelectric phonon modes with Lorentzian distributions. As shown in Fig. S7a, the 2^nd^ ZA can be well described with two Lorentzian distributions, corresponding to the lower and upper ZA phonon modes. Upon cooling, they abruptly decrease at about 200 K and disapper at about 50 K. High-order ZA phonon (indicated in Fig. S7b) also exhibits similir temperature-dependent behavior to 2^nd^ ZA phonon. Also shown in Fig. S7b are the fitting results for other modes, namely TO2, LO1 and A_2g_.

**S8. Temperature-dependent second harmonic generation in normal incidence**


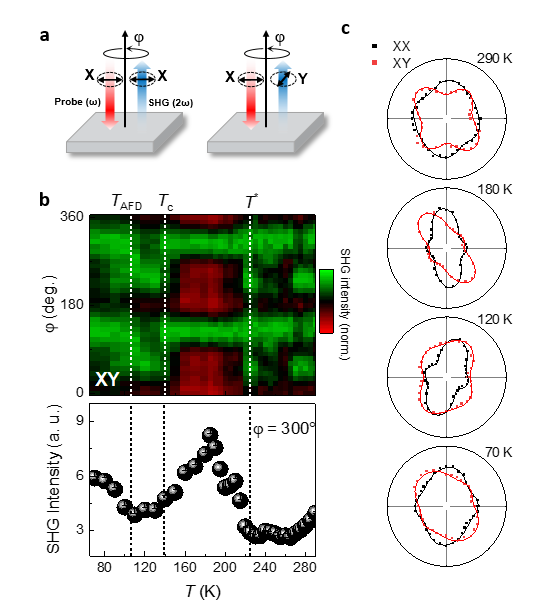


**Figure S8. Temperature-dependent SHG in the normal incidence for the [15|15]_4_ SL. (a)** Schematic of SHG experiment in the normal incidence. XX and XY represent parallel and orthogonal configuration, respectively. **(b)** Temperature-dependent normalized SHG results (top panel) and its intensity (bottom panel) in the XY configuration. **(c)** SHG polar patterns at four representative temperatures. Sold lines are the fitting results considering the monoclinic *m* symmetry.

We monitored the temperature-dependent structure changes in SLs using not only Raman spectroscopy, but also SHG measurement. Figure S8a shows the schematic of SHG experiment in the normal incidience. In this geometry, we can monitor the in-plane component of the dynamic polarization. We employed two different polarization configurations, parallel (XX) and orthogonal (XY) polarizations between input and SHG beams. Figure S8b displays the temperature-dependent normalized SHG anisotropy patterns and its intensity in the XY configuration. Both the SHG polar pattern and its intensity exhibt siginficant change at 100 K, 130 K and 230 K, denoted by *T*_AFD_, *T*_c_ and *T*^*^, respectively. It should be noted that *T*_AFD_ and *T*^*^ deterimined from the SHG measurement are in good agreement with those from the Raman measurement. As shown in Fig. S8c, the SHG signal at 290 K shows four-fold aniostopy polar patterns in both the XX and XY configurations. These polar patterns are well described by the theoretical line considering monoclinic *m* symmetry. Under cooling, this four-fold polar pattern is changed into two-fold-like at *T*^*^, accompanieed by an increase in the intensity. This demonstrates the enahancement of in-plane polarization component, originating from the formation of long-range polar order ^[4]^. Below *T*_c_, corresponding to the Curie temperature in the SRO layer, the polar pattern is slightly rotated without a noticable change in its intensity. This rotation of SHG polar pattern is usaually observed under the ferromagntic phase transition in various samples ^[5-7]^. We also observed the notable change in both the SHG polar pattern and the intensity at *T*_AFD_, corresponding to the transition temperature to the AFD phase with the 4*mm* tetrogonal symmetry ^[8]^.

**S9. Temperature-dependent thermal conductivity of SrRuO_3_/SrTiO_3_ superlattices**


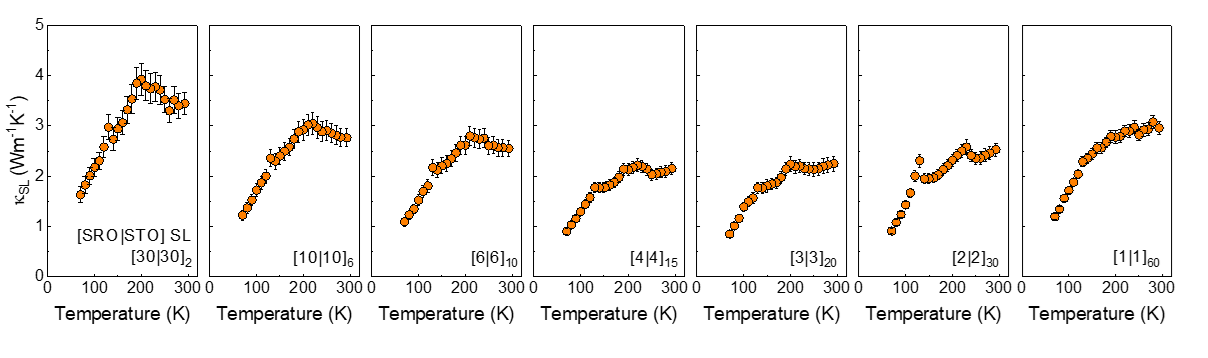


**Figure S9. Temperature-dependent κ of SLs for various thicknesses.**

In Fig. S9, we present full data of the temperature-dependent κ of SLs. Tracing κ at a given temperature as a function of the interface density, the coherent-incoherent crossover in the phonon transport is clearly manifested at all temperatures. For all SLs, κ decreases with decreasing the temperature, demonstrating that the boundary scattering is dominant in the SL. Notably, there clearly show anomalies at about 130 K and 230 K, which correspond to the ferromagnetic transition and the polar structural phase transition, respectively. Such anomalies are absent in the [1|1]_60_ SL. Although the intermixing between the SRO and STO layers might be relevant to this observation, the κ of the [1|1]_60_ SL is much larger than its alloy limit, and hence the intermixing effect can be excluded. Further investigations are required by considering the phase stabilities in the single-unit-cell limit of SRO and STO layers.

**S10. Temperature-dependent thermal conductivity of the SrTiO_3_ substrate**


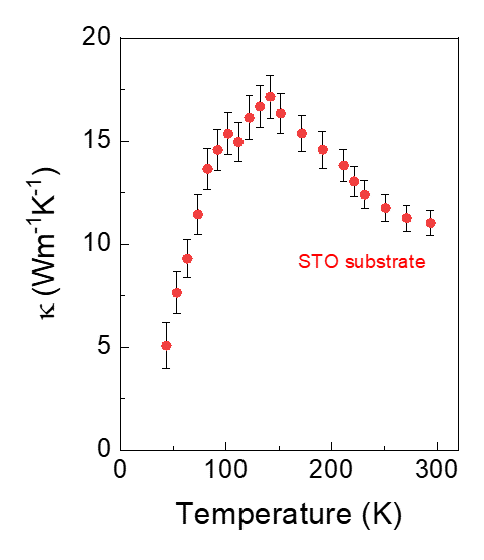


**Figure S10. Temperature-dependent κ of the STO substrate.**

Figure S10 presents the temperature-dependent κ of the STO substrate as a function of temperature. At room temperature, its value is 11.5 Wm^-1^K^-1^, which is similar to reference values ^[9]^. Whereas a hump structure is observed near 150 K, attributed to the AFD fluctuation, there is no notable change at the AFD transition temperature (~ 105 K).

**References**

1. S. Adachi, *J. Appl. Phys.* **1983**, 54, 1844.
2. M. V. Simkin, G. D. Mahan, *Phys. Rev. Lett.* **2000**, 84, 927.
3. M. N. Iliev, A. P. Litvinchuk, H. G. Lee, C. L. Chen, M. L. Dezaneti, C. W. Chu, V. G. Ivanov, M. V. Abrashev, V. N. Popov, *Phys. Rev. B* **1999**, 59, 364.
4. S. Checchia, M. Allieta, M. Coduri, M. Brunelli, M. Scavini, *Phys. Rev. B* **2016**, 94, 104201.
5. Y. Ogawa, Y. Kaneko, J. P. He, X. Z. Yu, T. Arima, Y. Tokura, *Phys. Rev. Lett.* **2004**, 92, 047401.
6. Y. Krockenberger, J. S. Lee, D. Okuyama, H. Nakao, Y. Murakami, M. Kawasaki, Y. Tokura, *Phys. Rev. B* **2011**, 83, 214414.
7. C. J. Roh, J. R. Kim, S. Park, Y. J. Shin, B. J. Yang, T. W. Noh, J. S. Lee, *Appl. Surf. Sci.* **2021**, 553, 149574.
8. S. Denev, A. Kumar, M. D. Biegalski, H. W. Jang, C. M. Folkman, A. Vasudevarao, Y. Han, I. M. Reaney, S. Trolier-McKinstry, C. B. Eom, D. G. Schlom, V. Gopalan, *Phys. Rev. Lett.* **2008**, 100, 257601.
9. V. Martelli, J. L. Jimenez, M. Continentino, E. Baggio-Saitovitch, K. Behnia, *Phys. Rev. Lett.* **2018**, 120, 125901.
